# Yolk sac tumor and dysgerminoma in the left gonad following gonadoblastoma in the right gonad in a 46,XY DSD with a novel SRY missense mutation: a case report

**DOI:** 10.1186/s12884-022-05317-3

**Published:** 2023-01-24

**Authors:** Chengxiu Xie, Jian Cai, Nan Li, Ping Hua, Zexuan Yang, Xia Yu, Dongmei Tang, Yu Hu, Qingsong Liu

**Affiliations:** 1 Department of Prenatal Diagnosis, Chengdu Women’s and Children’s Central Hospital, School of Medicine, University of Electronic Science and Technology of China, Chengdu, 611731 China; 2 Pathology Department, Chengdu Women’s and Children’s Central Hospital, School of Medicine, University of Electronic Science and Technology of China, Chengdu, 611731 China; 3 Ultrasound Department, Chengdu Women’s and Children’s Central Hospital, School of Medicine, University of Electronic Science and Technology of China, Chengdu, 611731 China; 4 Department of Medical Laboratory, Chengdu Women’s and Children’s Central Hospital, School of Medicine, University of Electronic Science and Technology of China, Chengdu, 611731 China; 5 Obstetrics and Gynecology Department, Chengdu Women’s and Children’s Central Hospital, School of Medicine, University of Electronic Science and Technology of China, Chengdu, 611731 China

**Keywords:** Disorders of sex development (DSD), Yolk sac tumor, Dysgerminoma, Gonadoblastoma (GB), Case report

## Abstract

**Background:**

Approximately 10–15% of 46,XY disorders of sex development (DSDs) have an SRY mutation residing in the high mobility group (HMG) domain. Here, we present a case of 46,XY DSD caused by a novel missense mutation in the HMG region of SRY rapidly progressing to germ cell tumors (GCTs).

**Case presentation:**

An adolescent female (15 years old) exhibiting primary amenorrhea was later diagnosed as a 46,XY female with bilateral gonadal dysplasia on the basis of peripheral lymphocyte karyotype 46,XY and a novel missense mutation in SRY (c.281 T > G, p.L94R). The novel missense mutation (c.281 T > G, p.L94R) and its adjacent region were conserved. Protein structure analysis showed that the mutant site was located in the middle of the HMG domain, and the mutant protein had a diminished ability to bind to DNA. Imaging examination revealed an adolescent female with a naive uterus. Laparoscopy and initial pathological examination revealed left gonadal dysplasia and right gonadal dysplasia with gonadoblastoma (GB). Right gonadectomy by laparoscopy was performed upon consent from the patient’s parents. Less than 1 year postoperatively, the left gonadal gland deteriorated as observed by the findings of a mass in the left adnexal region by pelvic MRI and serum AFP > 1000 ng/ml by serological tests, and then total hysterectomy and adnexal and left gonadectomy by laparoscopy were performed. The GCT stage was classified as stage Ic according to FIGO. At this time, pathologic examination showed that the left gonad had progressed to yolk sac tumor and dysgerminoma. The patient underwent chemotherapy post-operatively but developed type III myelosuppression and tumor recurrence several months later.

**Conclusions:**

The patient initially presented with right gonadoblastoma but chose only right gonadectomy by laparoscopy to preserve the female sex characteristics, which resulted in rapid deterioration of the left gonad and poor treatment outcomes. This case demonstrates the importance of early genetic diagnosis and treatment of 46,XY female DSD.

**Supplementary Information:**

The online version contains supplementary material available at 10.1186/s12884-022-05317-3.

## Background

46,XY female disorders of sex development (DSDs) with SRY mutations, historically known as Swyer syndrome, involve congenital incomplete or disordered gonadal development in which there is discordance between genetic, gonadal and phenotypic characteristics [1]. The SRY gene, located on p11.2 of the Y chromosome, has a sex-determining region and plays an essential role in the process of sex determination [2–4]. Mutations in the high mobility group (HMG) domain of the SRY gene affect binding to and bending of DNA or nuclear transport in approximately 10–15% of 46,XY gonadal dysplasia cases [5–8]. As a result, these mutations can lead to early errors in sex determination, which in turn prevents proper testicular formation.

46,XY DSD patients have an elevated risk for the development of germ cell tumors (GCTs) [9]. GCTs originate from primordial germ cells or gonocytes and can be subdivided into seminomas/dysgerminomas and nonseminomas with carcinoma in situ (CIS) or gonadoblastoma (GB) as precursor lesions [10, 11]. The risk of GCTs varies but is estimated to exceed 30% in patients with complete gonadal dysgenesis, and these tumors are usually bilateral [9]. The diagnosis and subdivision of GCTs are usually dependent on histopathology and immunohistochemistry. SOX9 and FOXL2 show mutual inhibition through β-catenin interaction with WNT4, thus promoting the development of the male and female reproductive systems, respectively [12]. OCT3/4, an octamer binding transcription factor, is consistently and specifically expressed in all GCTs with pluripotent potential, as well as in the testis of the neoplastic precursor CIS lesion and in the gonad of GB developed from DSD [13]. AFP is a characteristic immunohistochemical marker of yolk sac tumors, which may correlate with serum levels [14]. Moreover, some specific structures are helpful for subdividing GCTs, such as the Schiller-Duval (S-D) body [15] in yolk sac tumors and extensive calcification [16] in GB.

Here, we report a unique case of bilateral GB in an adolescent female presenting with primary amenorrhea at the age of 15 years who was initially diagnosed as 46,XY DSD and suffered rapid progression into yolk sac tumor and dysgerminoma in the left gonad following right gonadectomy by laparoscopy a year later. Mutation analysis identified a novel missense mutation (c.281 T > G, p.L94R) in the HMG domain of SRY, which resulted in compromised binding to DNA as determined by protein structure analysis. The first clinical diagnosis was left gonadal dysplasia and right gonadal dysplasia with GB based on pathological and imaging findings. The patient’s aspiration was to preserve her female characteristics, and her parents only consented to right gonadectomy by laparoscopy. Less than 1 year after the right gonadectomy, the left gonad deteriorated as observed by the findings of a mass in the left adnexal region by pelvic MRI and serum AFP > 1000 ng/ml by serological tests. Then, total hysterectomy and adnexal and left gonadectomy were performed by laparoscopy under general anesthesia. The final pathological report showed the formation of invasive seminoma (dysgerminoma, 20%) combined with an invasive nonseminoma component (yolk sac tumor, 80%). After chemotherapy, the patient developed type III myelosuppression and tumor recurrence several months later. To our knowledge, this is the first case describing a patient with a p.L94R mutation in SRY and underlines the importance of proper diagnosis. Although the patient obtained a definite diagnosis, the lesions still deteriorated rapidly because of failure to receive timely treatment of bilateral gonadectomy due to her personal aspiration of preserving female secondary sexual characteristics. This strongly suggests that early genetic diagnosis and treatment may prevent the development of invasive cancer in 46,XY DSD patients with an increased risk for GCTs.

## Case presentation

A 15-year-old senior high school female student (height: 168 cm, weight: 61 kg) was admitted first to Chengdu Women and Children’s Central Hospital with no menarche (Chengdu, China). The patient was born following a full-term normal delivery and had a birth weight of 3.25 kg. Her parents were nonconsanguineous to each other. The patient was her parents’ sole child, and her mother denied taking any sex hormone drugs or exposure to radioactive substances during her pregnancy. The parents also denied any family history. The patient exhibited a female appearance and voice, with little subcutaneous fat, no beard or Adam’s apple, bilateral breast B3, no tenderness, and several armpit hairs. Gynecological examination showed that the patient presented with normal female external genitalia but exhibited clitoral hypertrophy, urethral and vaginal openings located properly in the perineum area, no palpable or obvious mass in the bilateral groin and labia, and Tanner stage III pubic hair.

Transabdominal ultrasonography showed a naive uterus with a size of 4 cm × 1.6 cm and an endometrial thickness of 0.3 cm. The size of the left ovary was 1.9 × 1.0 cm, and that of the right ovary was 2.2 × 0.9 cm. Hysteroscopic examination of the right gonad revealed that it was olive shaped with a size of 3 × 2 × 1.5 cm. A pelvic MRI plain scan showed that the sizes of the uterine body and cervix were approximately 14.5 mm × 33 mm and 10 mm × 21 mm, respectively, and the visible endometrial thickness was 3 mm. The vagina was discontinuous, the middle and upper vagina were similar to a cord, the lumen was not observed, and the lower vagina was clear. Near the right lateral wall, there was an ovoid soft tissue signal with small dots of T2WI hyperintensity, approximately 21 mm × 13 mm in size.

Serum sex hormonal analysis revealed that the adrenocorticotropic hormone (ACTH) level was 28.40 pg/ml, the estradiol (E2) level was 42.86 pg/ml, the prolactin level was 12.41 ng/ml, the follicle stimulating hormone (FSH) level was 40.11 mIU/mL, the luteinizing hormone (LH) level was 20.45 mIU/mL, the progesterone level was 0.9 ng/ml, the 17-α-hydroxyprogesterone level was 3.99 mmol/L, the testosterone level was 86.18 ng/dl, and the anti-Mullerian hormone (AMH) level was 0.79 ng/mL. The hormonal results indicated that the patient had hypergonadotropic hypogonadism.

**Fig. 1:**
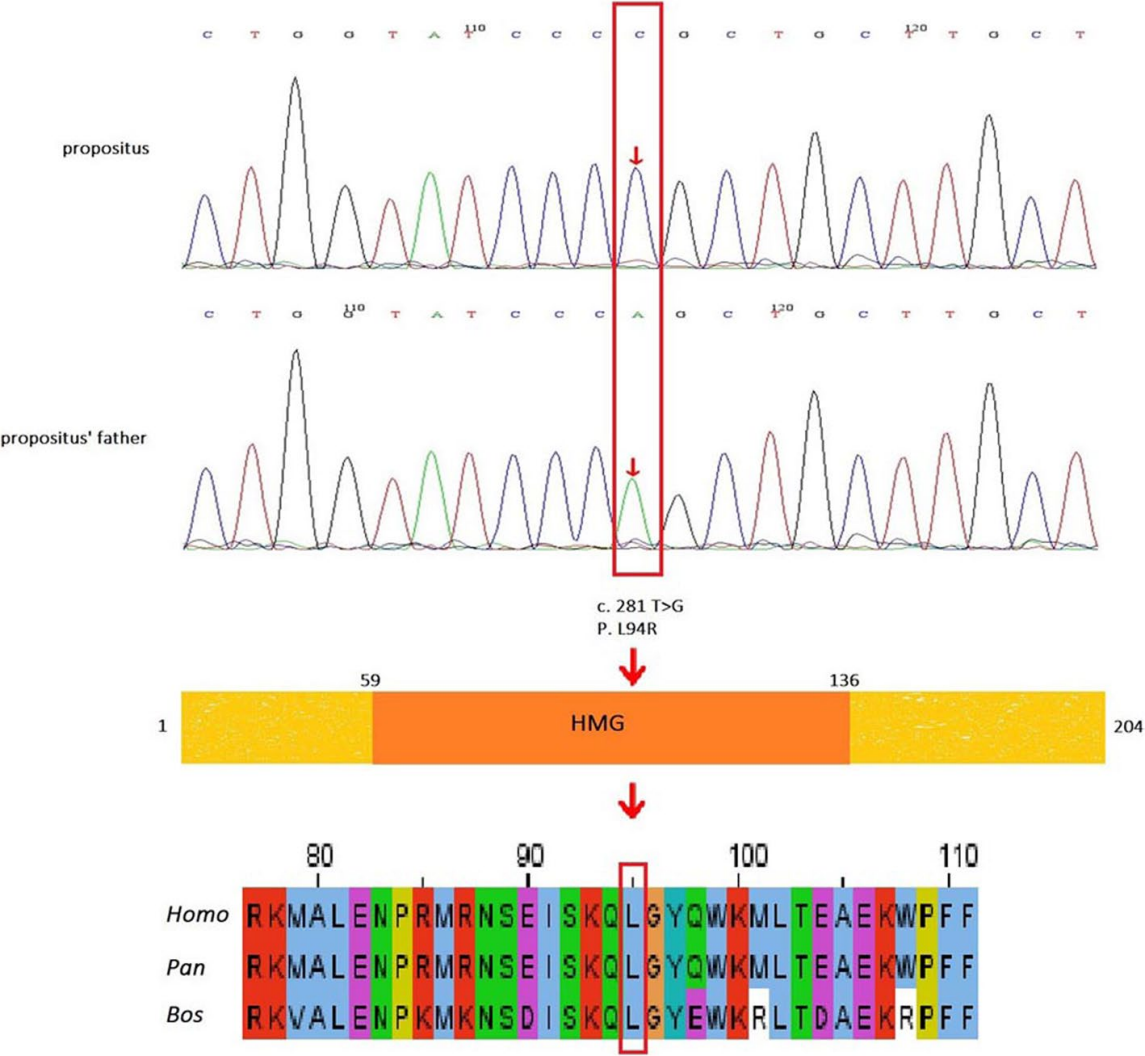
Schematic representation of the SRY protein, mutation location, and conservation. The propositus was detected to have a de novo hemizygotic mutation, c.281 T > G p.L94R, in exon 1 of SRY (the result of antisense chain sequencing is shown in the figure). Gene structure analysis indicated that this mutation occurred in the HMG domain, which is important for binding to and bending of DNA or nuclear transport. The affected amino acid and the surrounding region are conserved

**Fig. 2:**
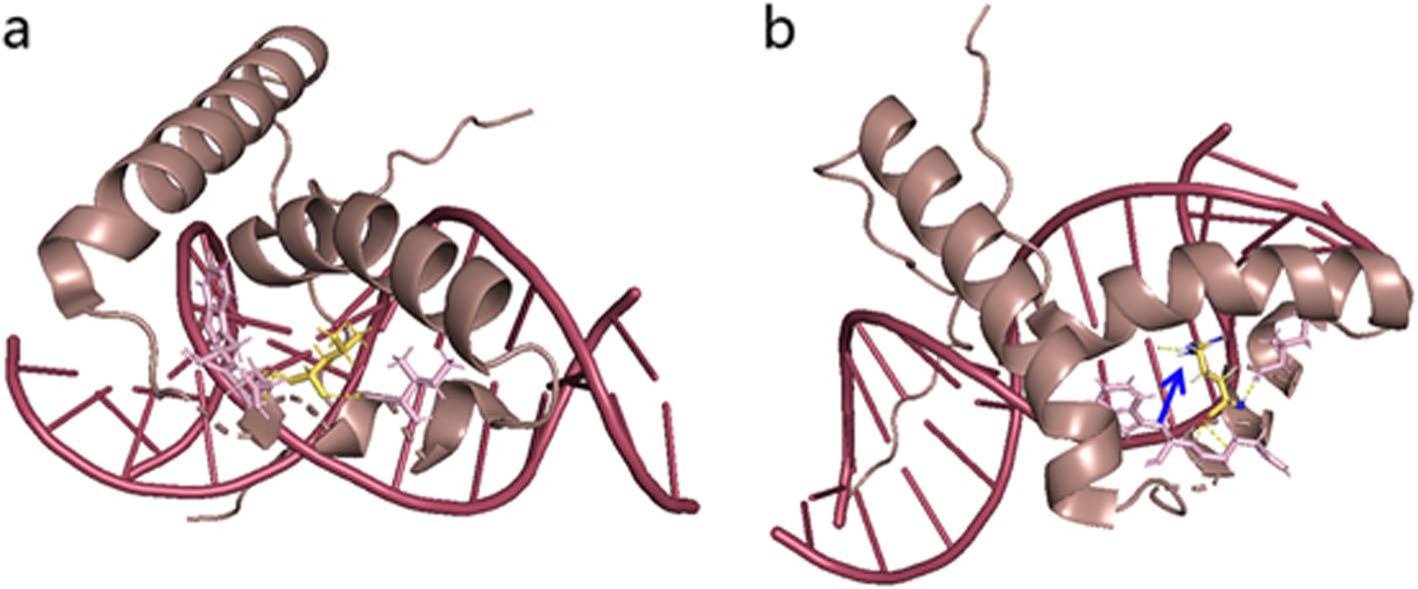
Structural illustration of the missense mutation in SRY. The mutation site in the wild-type (WT) SRY protein model (**a**) and mutant (MUT) SRY protein model (**b**) are shown in a and b. This mutation may affect the binding of SRY to the DNA duplex. We obtained the structure prediction of wild-type SRY protein from the Alphafold database. PyMOL Viewer software was used to generate the mutant SRY protein and visualize the effects of altered residues on protein-structure models. This single nucleotide mutation was predicted to change leucine to arginine at position 94. Importantly, the arginine in the mutated protein was predicted to generate a novel covalent bond. We assumed that the altered band may thus affect the function of SRY as a transcriptional regulator to recognize DNA double strands

The karyotype of peripheral blood was 46,XY. A novel missense mutation of c.281 T > G (p.L94R) in the SRY gene was detected by next-generation sequencing. The de novo mutation was confirmed by Sanger sequencing and pedigree analysis (Fig. 1). No pathogenicity variation of other gonadal-related genes was found. The L94 residue is highly conserved and located in the HMG box region, probably involved in DNA-binding specificity and stability (Fig. 2). Based on these results, the patient was initially diagnosed with 46,XY DSD with an SRY mutation.

**Fig. 3:**
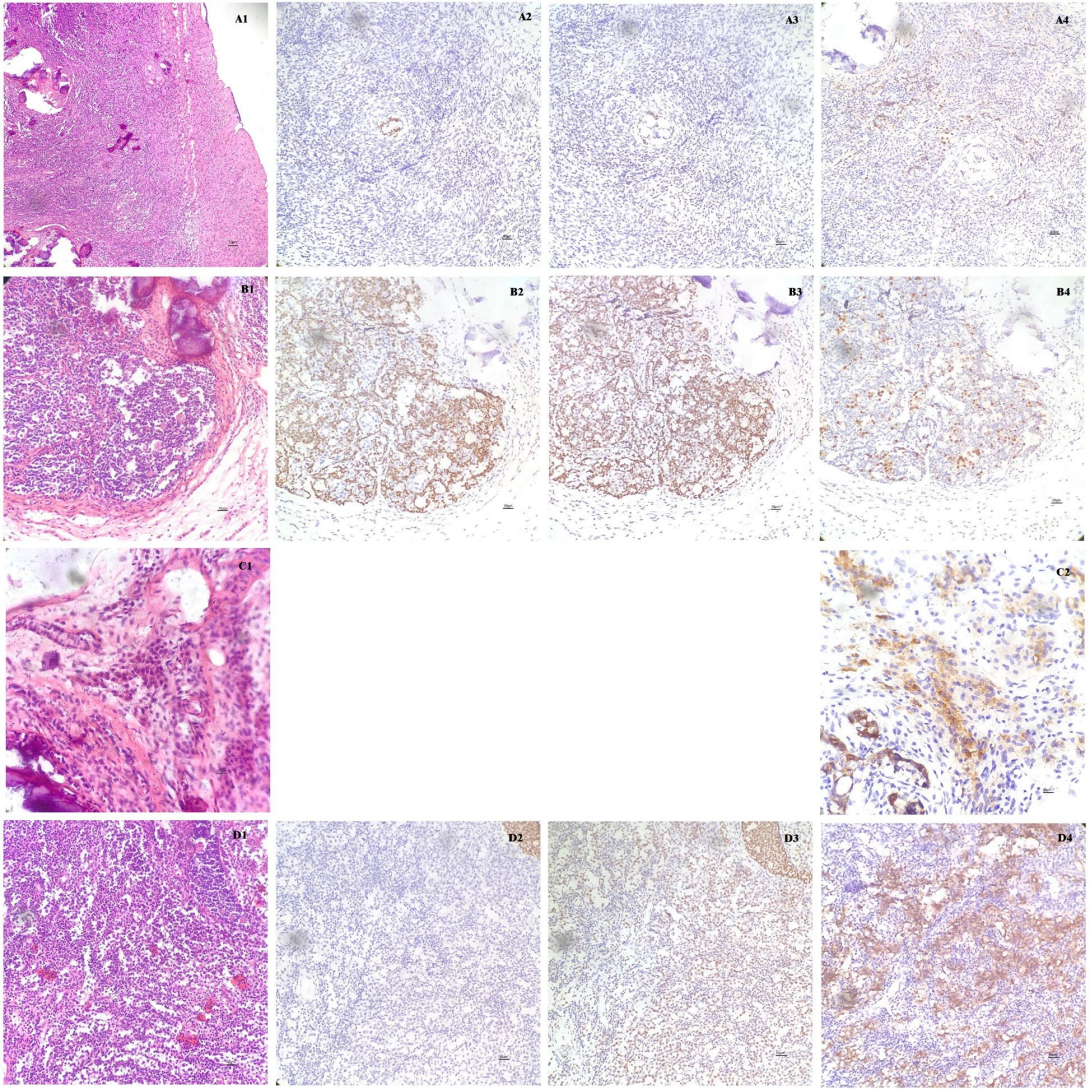
Histopathology of the right gonad. A1 Right primitive ovarian cortex. A2 FOXL2 was weakly expressed in the right primitive ovarian sex cord and stroma. A3 SOX9 was weakly expressed in the right primitive ovarian sex cord and stroma. A4 OCT4-positive primordial germ cells were scattered in the ovarian setting and were positive for both PLAP and CD117 (data not shown). B1 Gonadoblastoma in the right gonad (upright corner showing focal calcification). B2 The sex cord component of gonadoblastoma exhibited strong staining of FOXL2 and SOX9 (B3). B4 Groups of OCT4-positive primordial germ cells crowded adjacent to the sex cord of right gonadoblastoma, which implies clonal expansion. C1 Leydig cells in the right gonadal stroma. C2 The Leydig cells were α-inhibin positive. D1 Seminoma in the early stage. D2 There still remained a small quantity of FOXL2-positive sex cord components in the initiatory seminoma as well as SOX9-positive components (D3). D4 The neoplastic cells of seminoma exhibited CD117 positivity

The results of the first gonadal histopathology showed that there was a primitive ovarian cortex and several primitive follicular structures located in both gonads, and somewhere to the right of the ovary, Leydig cells were present. Both gonads presented as GB, with extensive calcification on the left; at the same time, invasive seminoma in its early stage was observed on the right. GB is made up of sex cord and primordial germ cells, which are marked by FOXL2 and SOX9 for the former and OCT4 for the latter. Furthermore, we could see groups of primordial germ cells crowded together, implying that clonal expansion probably occurred, and outside of the GB, the number of germ cells increased dramatically, which suggested the formation of invasive seminoma (Fig. 3). The upper, middle and lower poles of the left gonad had ovarioid stroma with no germ cells and extensive calcification, which suggested the existence of the “burn out” type of GB (data not shown). The clinical diagnosis was left gonadal dysplasia and right gonadal dysplasia with GB. Right gonadectomy was performed immediately after obtaining the consent of the patient’s parents. The serum testosterone level decreased to 2.73 ng/dL, which was consistent with the pathological findings, and the alpha-fetoprotein (AFP) level was < 0.5 ng/mL and carcinoembryonic antigen (CEA) level was 6.0 ng/mL after gonadectomy.

**Fig. 4:**
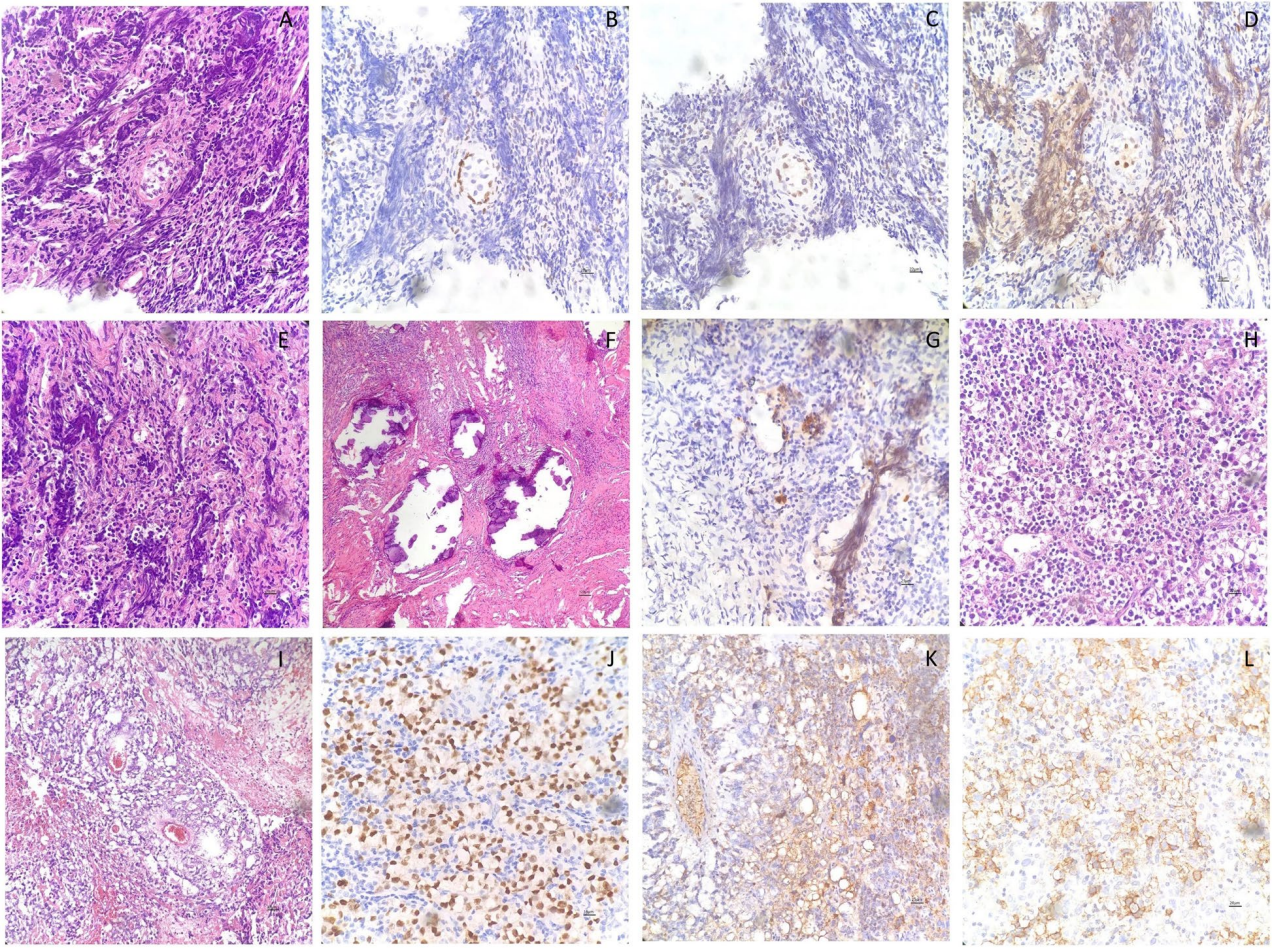
Histopathology of the left gonad. **A** A single follicular structure located in the cortex, suggestive of a primitive ovary. **B** FOXL2 was expressed more strongly at the epithelium of the follicular structure than SOX9 **(C)**. **D** A few OCT4-positive cells were present in the center of the follicular structure, suggestive of primordial germ cells. **E** UGT located in the left lower pole of the gonad. **F** Extensive calcification of the left upper pole prompted the diagnosis of “burn out” gonadoblastoma. **G** There were a few scattered OCT4-positive cells in the UGT at the left lower pole. **H** The invasive seminoma. **I** Yolk sac tumor showing the S-D body. **J** The neoplastic cells of seminoma exhibited OCT4 positivity as well as that of D2–40 **(L)**. **K** Yolk sac tumor exhibited AFP positivity

One year later, a left gonadal malignancy was observed by the findings of a mass in the left adnexal region by pelvic MRI and a serum AFP level exceeding 1000 ng/ml; the testosterone level increased to 15.9 ng/dL. Then, total hysterectomy and adnexal and left gonadal resection were performed by laparoscopy under general anesthesia after obtaining the consent of the patient’s parents. The AFP level decreased to 9.5 ng/mL after the operation. The histopathology results showed mixed germ cell tumors made up of 80% yolk sac tumors and 20% invasive seminoma in the excised left mass. The yolk sac tumor component displayed the specific structure of the S-D body and strongly positive staining for AFP and OCT4. In addition, positive staining of OCT4 and D2–40 was observed in the neoplastic cells of the invasive seminoma component. Furthermore, undifferentiated gonadal tissue (UGT) was also observed in the remaining left gonad (Fig. 4).

The patient underwent 4 cycles of the BEP chemotherapy regimen (bleomycin 15 U/m^2^, etoposide 100 mg/m^2^ and cis-platinum 20 mg/m^2^) after the operation. After chemotherapy, the patient presented with grade III myelosuppression. Six months after discharge, the patient dictated on a telephone follow-up that her tumor had recurred and she was undergoing further treatment at another hospital.

## Discussion and conclusions

Mutated SRY is one of the main pathogenic genes of 46,XY DSD. Here, c.281 T > G (p.L94R), a novel mutation of SRY in a 46,XY adolescent female, was detected by high-throughput sequencing. p.L94R is located at the same mutation loci as p.L94P reported previously [17]. Bioinformatics was performed, and p.L94R was presumed to be a new pathogenic mutation. The suspected etiology for the 46,XY DSD phenotype caused by this mutation may be that the mutant protein reduced the specificity and stability of DNA binding.

The patient in this study had a typical genetic phenotype and gonadal phenotype inconsistency, as described in previously reported 46,XY DSD patients [18]. In addition to the development of secondary sexual characteristics and the level of sex hormones, primitive ovarian cortex was observed in the bilateral gonads detected by histopathology in this case. Because of the absence of proper SRY expression by inactivating mutations of p.L94R, stromal cells will not differentiate into Sertoli cells and instead switch to the female pathway to form the primitive ovarian cortex [19]. This was demonstrated by the immunoreaction pattern of FOXL2, SOX9, and OCT4 in the bilateral gonads. The female differentiation pathway is initiated after activating the appropriate expression of FOXL2 and many other genes, resulting in the differentiation of stromal cells into granulosa cells [20]. Leydig cells were also found by histological testing of the right gonad. A subset of stromal cells will differentiate into Leydig cells following the male differentiation pathway [21]. These results fully illustrate the patient’s gonadal dysplasia.

The patient was initially diagnosed with 46,XY female DSD with simple gonadal dysplasia and bilateral unclear gonadal masses based on genetics, sex hormones, imaging and phenotype. It has been reported that GB occurs almost entirely in the dysgenetic gonads of an individual who has a disorder of sex development involving the Y chromosome [22]. The patient was diagnosed with bilateral gonad GB by further histopathology. However, nearly 1 year later, the patient developed an abnormal increase in AFP and left gonadal malignancy with dysgerminoma and yolk sac tumor.

The patient in this study chose only right gonadectomy after gonadoblastoma, and initial invasive seminoma was detected on the right gonad (no infiltrating lesions were detected on the left side at this time) to preserve theoretical female sexuality, resulting in deterioration of the left gonad in the short term. We learned through a telephone follow-up that the patient’s cancer recurred (which side or both is unknown) after left gonadectomy. As seminoma on the right gonad showed in the early stage, the possible reason may be the rapid accumulation of abnormally expressed SRY and its linked proteins in the left gonad after right gonadectomy [23]. Considering that the risk of malignant transformation of germ cells in 46,XY DSD patients is highly heterogeneous [24], the underlying crucial factors involved in maintaining the germ cells in an undifferentiated and possibly proliferative state remain to be further investigated.

## Conclusion

The patient initially presented with a right gonadoblastoma but chose only right gonadectomy by laparoscopy to preserve her female characteristics, which resulted in rapid deterioration of the left gonad with yolk sac tumor and dysgerminoma, poor treatment outcome with type III myelosuppression and tumor recurrence. This case demonstrates the importance of early genetic diagnosis and treatment of 46,XY female DSD.

## Supplementary Information

**Additional file 1.**

## Data Availability

All data generated or analyzed during this study are included in this published article. The next generation sequencing and Sanger sequencing were done by third-party laboratories, which can only provide final results (see Supplementary figure), not raw data.

## Abbreviations

DSD: Disorders of sex development
HMG: High mobility group
GCTs: Germ cell tumors
CIS: Carcinoma in situ
GB: Gonadoblastoma
S-D: Schiller-Duval
AMH: Anti-Mullerian hormone
AFP: Alpha-fetoprotein
CEA: Carcinoembryonic antigen
UGT: Undifferentiated gonadal tissue
