# Supplementary figures and images for: Yolk sac tumor and dysgerminoma in the left gonad following gonadoblastoma in the right gonad in a 46,XY DSD with a novel SRY missense mutation: a case report
Source: BMC Pregnancy Childbirth. 2023 Jan 24;23:58. doi: 10.1186/s12884-022-05317-3 (PMC9872390; doi:10.1186/s12884-022-05317-3)

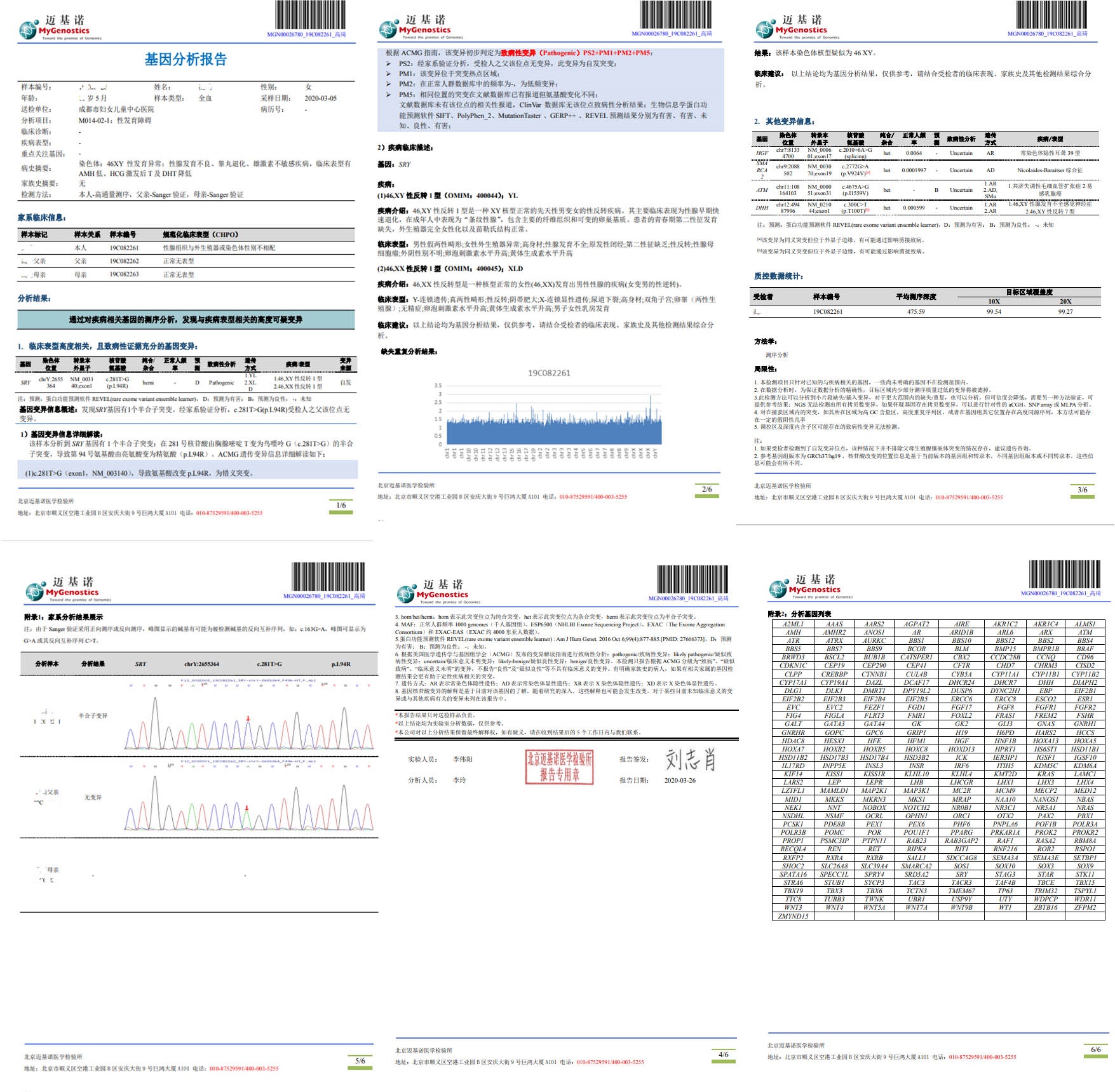

Supplement: Supplementary file 1 — Additional file 1. [file 12884_2022_5317_MOESM1_ESM.jpg]
